# The Amphibian Antimicrobial Peptide Temporin B Inhibits *In Vitro* Herpes Simplex Virus 1 Infection

**DOI:** 10.1128/AAC.02367-17

**Published:** 2018-04-26

**Authors:** M. E. Marcocci, D. Amatore, S. Villa, B. Casciaro, P. Aimola, G. Franci, P. Grieco, M. Galdiero, A. T. Palamara, M. L. Mangoni, L. Nencioni

**Affiliations:** a Department of Public Health and Infectious Diseases, Sapienza University of Rome, and Istituto Pasteur Italia-Fondazione Cenci Bolognetti, Rome, Italy; b Department of Biochemical Sciences, Sapienza University of Rome, and Istituto Pasteur Italia-Fondazione Cenci Bolognetti, Rome, Italy; c Department of Life, Health and Environmental Sciences, University of L'Aquila, L'Aquila, Italy; d Department of Experimental Medicine, University of Study of Campania Luigi Vanvitelli, Naples, Italy; e Interuniversity Research Centre on Bioactive Peptides (CiRPEB), University of Naples Federico II, Naples, Italy; f Department of Pharmacy, University of Naples Federico II, Naples, Italy; g IRCCS, San Raffaele Pisana, Telematic University, Rome, Italy

**Keywords:** HSV-1, temporin, virucidal, antimicrobial peptide, antiviral agents

## Abstract

The herpes simplex virus 1 (HSV-1) is widespread in the population, and in most cases its infection is asymptomatic. The currently available anti-HSV-1 drugs are acyclovir and its derivatives, although long-term therapy with these agents can lead to drug resistance. Thus, the discovery of novel antiherpetic compounds deserves additional effort. Naturally occurring antimicrobial peptides (AMPs) represent an interesting class of molecules with potential antiviral properties. To the best of our knowledge, this study is the first demonstration of the *in vitro* anti-HSV-1 activity of temporin B (TB), a short membrane-active amphibian AMP. In particular, when HSV-1 was preincubated with 20 μg/ml TB, significant antiviral activity was observed (a 5-log reduction of the virus titer). Such an effect was due to the disruption of the viral envelope, as demonstrated by transmission electron microscopy. Moreover, TB partially affected different stages of the HSV-1 life cycle, including the attachment and the entry of the virus into the host cell, as well as the subsequent postinfection phase. Furthermore, its efficacy was confirmed on human epithelial cells, suggesting TB as a novel approach for the prevention and/or treatment of HSV-1 infections.

## INTRODUCTION

One of the most widespread human pathogens is the herpes simplex virus 1 (HSV-1), an enveloped DNA virus whose primary infection generally occurs in the oropharyngeal mucosa, followed by neuronal latency in the peripheral ganglia. Reactivation of the latent results either in asymptomatic virus shedding or in recurrent herpes disease (1–3). In most cases, HSV-1 infection is asymptomatic, and the majority of people are unaware of their HSV-1 status. However, in immunocompromised individuals, HSV-1 recurrences are associated with severe symptoms and, rarely, continuous virus reactivations can be the beginning of more serious complications, such as encephalitis and keratitis (4). HSV-1 infections are usually treated with antiviral agents such as acyclovir and its derivatives; unfortunately, these therapies neither prevent recurrence arising from the reactivation of the latent virus, nor do they completely eliminate the virus (5). Among immunocompetent patients, HSV-1 infection is controlled rapidly by the human host's immune system, thus requiring short-term antiviral therapy with minimal risk of developing resistance. In contrast, immunocompromised patients may be unable to control HSV-1 infection and generally need long-term antiherpetic therapy, which is more likely to induce drug resistance (6, 7). In this context, it is essential to develop novel anti-HSV-1 agents.

Recently, naturally occurring antimicrobial peptides (AMPs) have been studied for their potential antiviral properties, as demonstrated against several viruses (8–11). AMPs are expressed in almost all living organisms, including plants and animals, as conserved products of innate immunity (12). Frog skin is one of the richest sources of AMPs that are synthesized by adrenergically innervated dermal glands and stored within granules that are released onto the skin surface by a holocrine mechanism in response to alarm or injury (13, 14). A family of amphibian AMPs, originally isolated from the European red frog Rana temporaria, is called temporins. These peptides represent the shortest AMPs found in nature to date with a typical length of 10 to 13 amino acids and a conserved sequence (15, 16). They are amidated at the C terminus and are characterized by a weak cationic charge (net charge ranging from +2 to +3) owing to the presence of only one or two positively charged amino acids, such as lysine or arginine, in their sequence.

Temporins, like other AMPs, exert antimicrobial activity against bacteria and mycetes (17, 18), while their antiviral activity has been reported only against a few enveloped or nonenveloped viruses of ectothermic animals (19). Furthermore, no studies regarding the activity of temporins against human viruses have been reported to date.

Here, we demonstrate for the first time the *in vitro* antiviral activity of temporin B (TB) against HSV-1. When added to HSV-1-infected cells, TB significantly reduced the virus titer, but, more importantly, the greatest inhibition was obtained by preincubation of HSV-1 with the peptide for 1 h at 37°C, thus demonstrating its virucidal activity.

## RESULTS

### Temporin B is not cytotoxic and reduces the HSV-1 titer.

**FIG 1:**
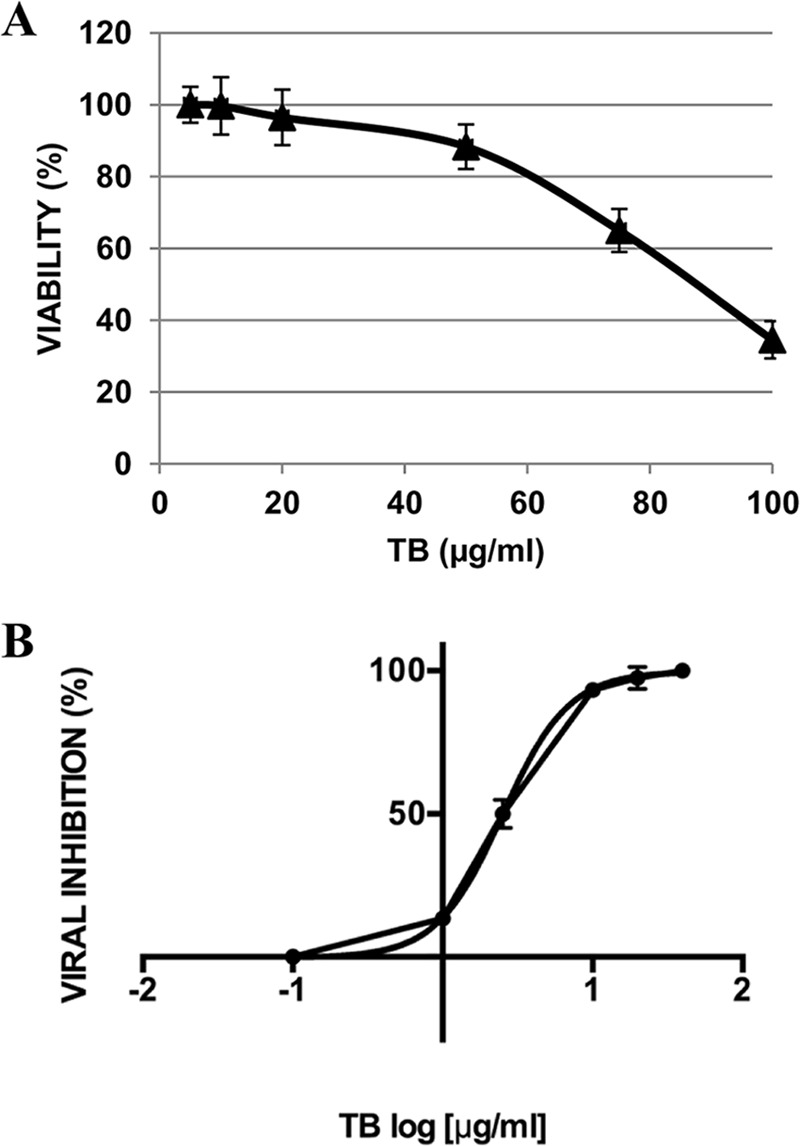
(A) Cytotoxic (CC_50_) and (B) antiviral (IC_50_) effects of TB on Vero cells. (A) Cell monolayers were incubated with increasing concentrations of the compound for 24 h, and cell proliferation was determined by an MTT assay. The CC_50_ of TB was calculated by regression analysis of the dose-response curve. (B) HSV-1-infected cells were treated with the peptide at concentrations ranging from 0.1 to 40 μg/ml, and viral titer inhibition was calculated by plaque reduction assay. The IC_50_ of the compound was calculated by regression analysis as described in Materials and Methods. Values are expressed as means ± the SD from three experiments, each performed in duplicate.

In a first set of experiments, we evaluated the effect of TB on Vero cell viability. To do this, cells treated with different concentrations (1 to 100 μg/ml) of peptide for 24 h were stained with trypan blue; their microscopic examination revealed no significant mortality in cells treated with the peptide at a concentration up to 40 μg/ml. In contrast, at higher concentrations, morphological alterations, loss of cell viability, and modification of the cell multiplication rate were observed (data not shown). These results were confirmed by means of an MTT [3-(4,5-dimethyl-2-thiazolyl)-2,5-diphenyl-2*H*-tetrazolium bromide]-based assay. As shown in Fig. 1A, cell proliferation was not affected by TB at low doses, whereas it decreased in a dose-dependent manner starting from 50 μg/ml. The 50% cytotoxic concentration (CC_50_) for the peptide was 90.4 μg/ml. On the basis of these results, in order to evaluate the potential antiviral activity of TB against HSV-1, we used the peptide at concentrations ranging from 0.1 to 40 μg/ml. To do this, a plaque reduction assay was performed. The results (Fig. 1B) indicate that TB reduced the HSV-1 titer in a dose-dependent way. The 50% inhibitory concentration (IC_50_) was 2.507 μg/ml (95% confidence interval = 2.325 to 2.703; selectivity index [CC_50_/IC_50_] = 36.06). On the basis of the obtained results, the next experiments were performed using TB at 20 μg/ml.

### TB affects life cycle and cell to cell spreading of HSV-1.

**FIG 2:**
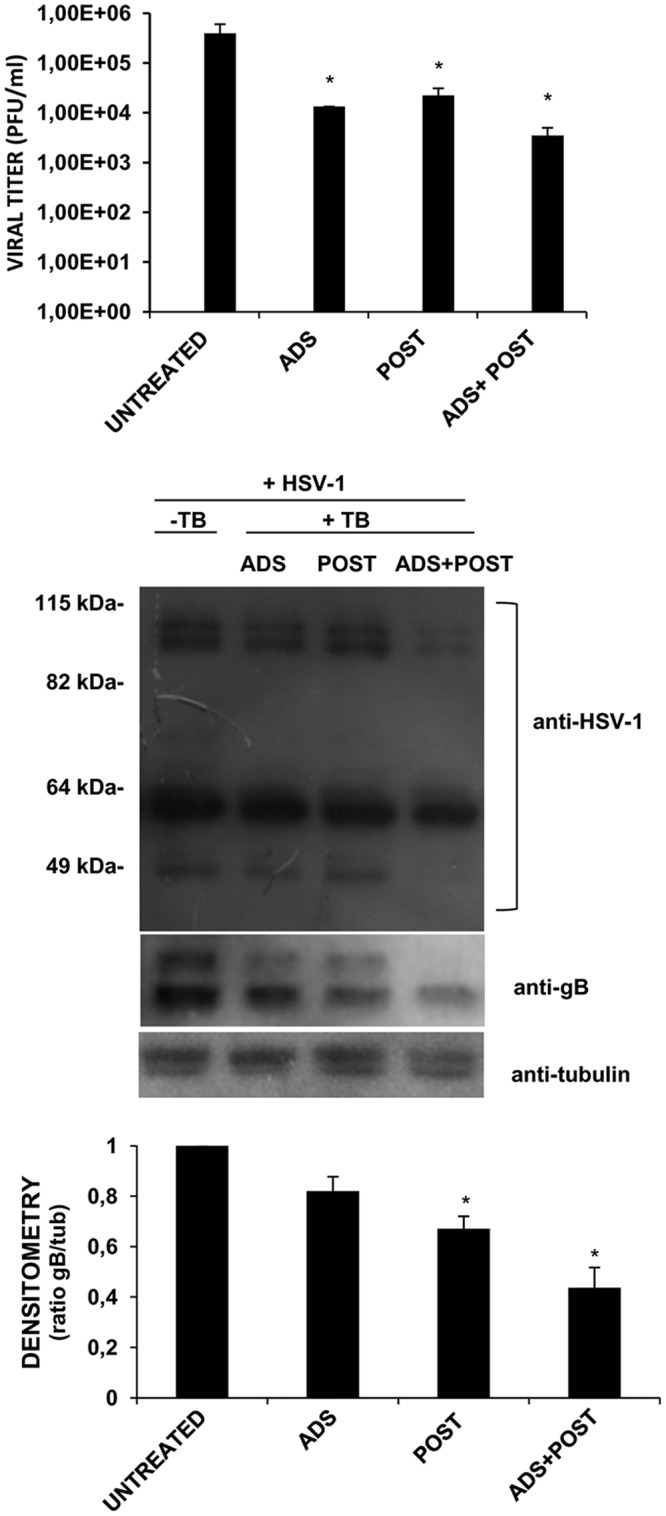
TB reduces HSV-1 replication at different stages of the virus life cycle. A time-of-addition assay was performed. TB was added to Vero cells as a single dose (during adsorption [ADS] or after adsorption for the following 24 h [POST]) and as double doses (ADS+POST). Supernatants were subjected to a standard plaque assay to evaluate viral production (upper panel). Data are expressed as means ± the SD from four independent experiments, each performed in duplicate (*, *P* < 0.05 versus untreated sample). Cell lysates were analyzed by Western blotting with anti-HSV-1 and anti-gB antibodies; tubulin was used as a loading control (lower panel). Densitometric analysis of gB levels is shown in the graph under the representative Western blot, and data are expressed as means ± the SD from three independent experiments (*, *P* < 0.05 versus untreated cells).

To evaluate the effect of TB on HSV-1 life cycle, Vero cells were infected with HSV-1 and incubated with TB at different times as follows: (i) during the viral adsorption (1 h [ADS]), (ii) immediately after viral adsorption (for 24 h [POST]), and (iii) during viral adsorption and for the following 24 h (ADS+POST). The results showed that TB was effective in reducing HSV-1 titer of ∼1.5 log under all of the experimental conditions tested (Fig. 2, upper panel). These results were confirmed through the analysis of infected cell proteins (ICPs) and late structural viral proteins, including glycoprotein B (gB). As shown in Fig. 2 (lower panel), the viral protein content was reduced in TB-treated cells under any condition with respect to untreated infected cells. Interestingly, the highest inhibition was obtained when the peptide was added as double doses (ADS+POST), as confirmed by densitometric analysis of gB (an ∼2.5-fold reduction).

**FIG 3:**
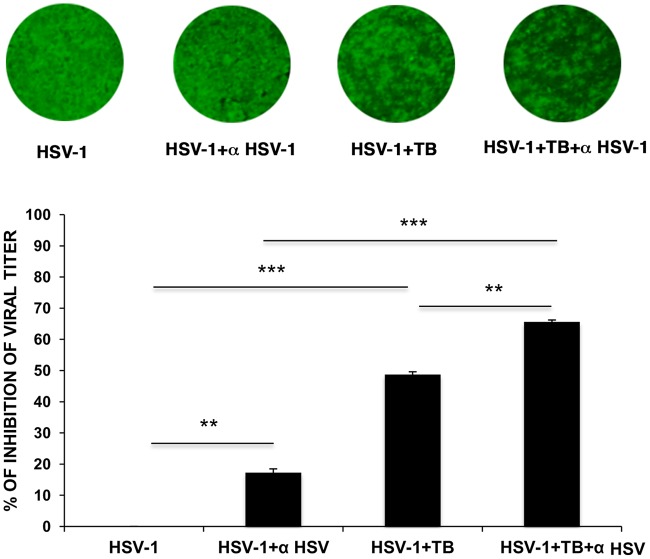
TB inhibits HSV-1 cell-to-cell spread. Vero cells were seeded in 96-well plates, infected with HSV-1 (MOI = 0.1), and treated or not treated with TB during and after viral adsorption. Anti-HSV-1 antibody (α HSV-1) at 10 μg/ml was added for 24 h postinfection. Foci of infection were visualized by immunostaining with mouse anti-gB and IRDye 800CW goat anti-mouse antibodies. A representative image of one of three experiments is shown in the upper panel. The relative fluorescence intensity was detected with the LI-COR Odyssey infrared imaging system, quantified using LI-COR Image Studio Software, and reported in the graph (in the lower panel) as the percent viral titer inhibition (**, *P* < 0.01; ***, *P* < 0.001).

On the basis of these results, we evaluated the effect of TB on HSV-1 cell-to-cell spread. Indeed, it is known that gB, gD, gH, and gL are the principal HSV-1 glycoproteins required for virus entry into the cell and for cell-to-cell spread (20). To assess this effect, confluent monolayer of Vero cells were treated or not with TB during viral adsorption and for the following 24 h of infection. Moreover, some samples were also incubated with HSV-1 neutralizing antibody to ensure that the occurred HSV-1 infection was due to cell-to-cell spread. Immunostaining analysis using a LI-COR Odyssey infrared imaging system (Fig. 3) revealed that neutralizing antibody and TB were able to reduce the foci of infection by about 20 and 50%, respectively, compared to untreated infected cells. Importantly, in the presence of both antibody and TB the inhibition was higher with respect to that obtained by single treatment (about 65% [*P* < 0.001 versus untreated infected cells]), suggesting that TB may prevent the cell-to-cell spreading of HSV-1.

### TB inhibits the attachment of the virus to host cell.

**FIG 4:**
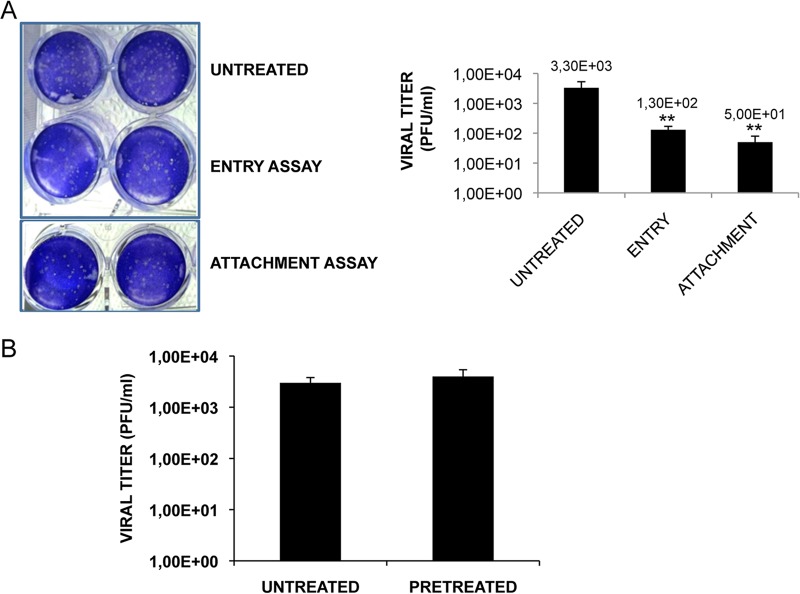
TB inhibits HSV-1 attachment to the host cells. (A) In the attachment assay, Vero cells were infected with a combination of HSV-1 plus TB, and inoculated cultures were kept for 1 h at 4°C to allow virus attachment but not entry. A plaque reduction assay was then performed. In the entry assay, cells were infected and kept at 4°C for 1 h to allow virus attachment. After a washing step, TB was added to the cells, and the cultures were incubated for 1 h at 37°C to allow HSV-1 penetration. After a single wash with PBS (pH 3) to remove eventually attached virus particles from the cell surface, the cells were subjected to a plaque reduction assay. Viral titers, expressed as PFU/ml in the graph, are means ± the SD from three independent experiments, each performed in duplicate (**, *P* < 0.01 versus untreated samples). For plaques stained with crystal violet, the results of one representative experiment of three performed are shown. (B) Vero cells were pretreated with the peptide for 3 h at 37°C before HSV-1 infection. Conditioned medium samples were collected 24 h after infection and subjected to a standard plaque assay. Data are means ± the SD from four experiments, each performed in duplicate.

Next, we investigated the antiviral activity of TB during the very early phases of HSV-1 life cycle, i.e., the attachment and penetration phases. To this aim, two different tests were performed: (i) an attachment assay, during which the virus can only bind the surface of the host cells but does not enter the cell, in the presence or absence of TB, and (ii) an entry assay, during which the peptide was added immediately after virus attachment to assess its ability to prevent entry. As shown in Fig. 4A, the HSV-1 titer was significantly reduced by about 2 logs and 1 log in the attachment and entry assays, respectively. To confirm that the antiherpetic efficacy of TB was greater during the attachment phase of virus life cycle and to exclude the possibility that TB could alter the binding of the virus to the host cell by interfering with some receptors on the cell surface, cell monolayers were pretreated with the peptide for 3 h at 37°C before HSV-1 infection. Supernatants of infected cells were recovered 24 h after infection and used for standard plaque assay. No difference in viral replication between untreated and TB-pretreated cells was observed (Fig. 4B), suggesting that TB does not exert any activity on the host cell surface.

### TB possesses virucidal properties.

**FIG 5:**
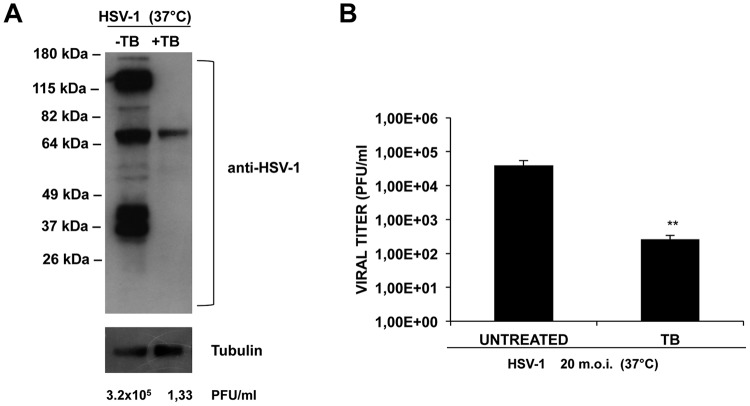
TB exerts a virucidal effect. (A) HSV-1 (MOI = 1) was preincubated with TB at 37°C for 1 h, and the mixture was used to infect Vero cells for 24 h. Infected cellular monolayers were used for a Western blot assay, which was performed with an HSV-1 antibody. Supernatants were collected, and their viral content was quantified as PFU/ml by a standard plaque assay, as reported under the Western blot image. (B) HSV-1 (MOI = 20) was preincubated with TB at 37°C for 1 h, and the mixture was diluted 20-fold (to obtain an MOI of 1) before infection. A standard plaque assay was performed. Data are means ± the SD from four experiments, each performed in duplicate (**, *P* < 0.01 versus untreated cells).

To verify the potentially direct effect of TB on virions, HSV-1 was preincubated with TB (HSV-1+TB) or no TB (HSV-1) for 1 h at 37°C, and then the mixtures were used to infect the cells. Immediately after virus adsorption, the cells were washed, followed by incubation for 24 h with fresh medium. The supernatant of the infected cells was used to perform a standard plaque assay, while the cell monolayer was harvested and subjected to Western blot analysis of viral proteins. Surprisingly, the plaques count showed that TB reduced by 5 log the HSV-1 titer compared to the untreated infected cells (Fig. 5A, below the gel). This strong reduction corresponds to 99.99% inhibition of HSV-1 replication, suggesting that TB acts directly on viral particles. These data were also confirmed by the almost complete absence of viral proteins in HSV-1+TB samples compared to HSV-1 samples (Fig. 5A).

To confirm the direct effect of TB on HSV-1 particles, a high multiplicity of infection (MOI) of HSV-1 (20-fold higher than the standard conditions, i.e., 20 MOI) were preincubated with 20 μg/ml TB at 37°C for 1 h. The mixture was then diluted to 1 MOI and used to infect cells. After viral adsorption, the cells were incubated with fresh medium supplemented with carboxymethyl cellulose (CMC), and a plaque reduction assay was performed. As shown in Fig. 5B, although in this assay the TB/HSV-1 ratio was altered compared to standard experimental procedures, the peptide was able to significantly reduce plaque formation (>2-log inhibition). These results support a direct effect of TB on HSV-1 particles that is probably due to its interaction with some components of the viral envelope and/or to disruption of the external viral layer (virucidal effect).

**FIG 6:**
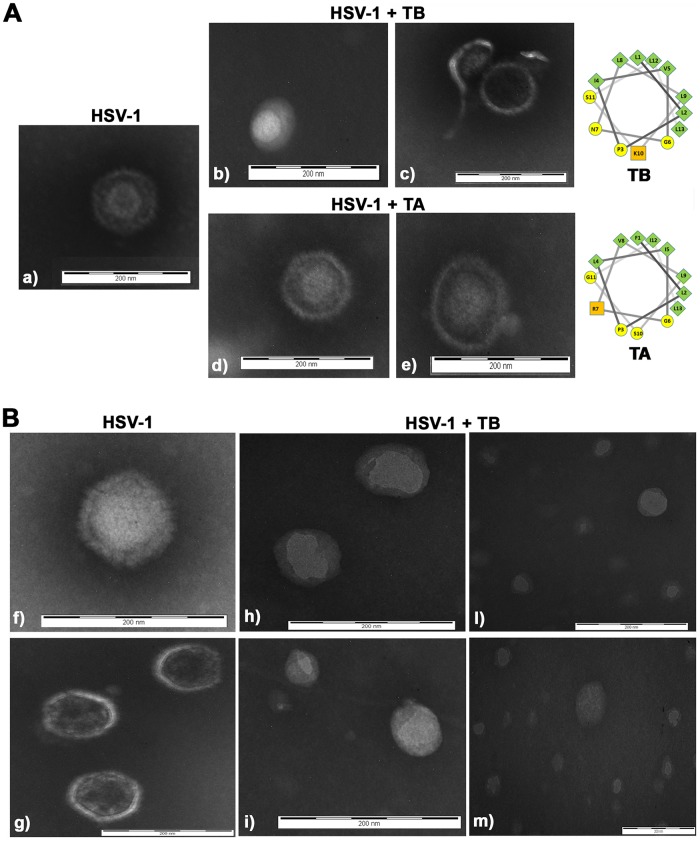
TB disrupts the HSV-1 envelope. (A) TB or TA peptides were incubated with HSV-1 (1 × 10^6^ PFU) virions for 1 h at 37°C as described in Materials and Methods. The morphology of the viral particles was analyzed by TEM and compared to that of untreated particles. Images were saved at ×130,000 magnification; (B) HSV-1 (5 × 10^6^ PFU) was incubated with TB as described previously. Images were saved at ×180,000 magnification. The images are representative of results obtained from duplicates of two independent experiments. Helical wheel projections of TB and TA are also shown (green, yellow, and orange symbols refer to nonpolar, polar uncharged, and basic residues, respectively).

To verify this hypothesis, we decided to study the morphology of virus particles (incubated or not with TB) by transmission electron microscopy (TEM). In Fig. 6Aa, the image of HSV-1 is shown. All viral components (envelope, tegument, and nucleocapsid) were clearly visible and intact. In contrast, in TB-pretreated HSV-1 virions a loss of viral envelope integrity is evident (Fig. 6Ab and c), confirming the virucidal effect of TB on HSV-1 virions. Afterward, to check whether such virucidal effect was an exclusive property of TB, we tested the activity of another member of the temporins family, i.e., TA (FLPLIGRVLSGIL-NH_2_; Selleck Chemicals, Houston, TX) for comparison. Note that TA previously showed activity against different viruses (19). Like TB, TA is also a peptide with an alpha-helical structure in membrane-mimicking environments and has a net charge of +2 at neutral pH and a length of 13 amino acids (Fig. 6) (16). TA was able to interfere with HSV-1 replication when it was preincubated with the viral particles at 37°C for 1 h, but this inhibition was lower than that of TB (1 log versus 5 log) (data not shown). Importantly, when the effect of TA on viral particles was visualized by electron microscopy, no disruption of the viral envelope was detected (Fig. 6Ad and e). Finally, to confirm whether TB was able to disrupt the envelope of the virus, we decided to incubate more viral particles with TB and quantified the virions that were broken by the peptide (Fig. 6B). Many TB-pretreated HSV-1 virions lost or showed a broken envelope (ca. 88.8% ± 7.6% or ∼8.2% ± 5.3%, respectively), whereas ca. 2.9% ± 2.3% of the virions were still intact (*P* < 0.05, intact versus disrupted envelope). All of these results strongly indicate that TB is effective in inhibiting viral replication by disrupting HSV-1 envelope.

### TB reduces HSV-1 titer in human epithelial cells.

**FIG 7:**
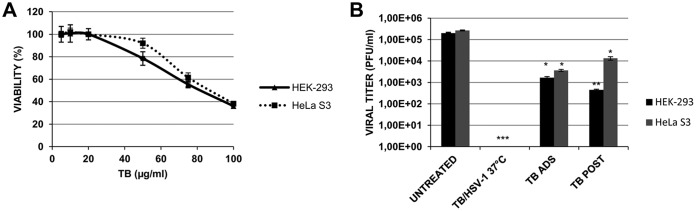
Cytotoxic (CC_50_) (A) and antiviral (B) effects of TB on human cells. (A) Confluent monolayers of HeLa S3 and HEK-293 cells were incubated with increasing concentration of TB for 24 h, and cell proliferation was determined by an MTT assay. The TB CC_50_ was calculated by regression analysis of the dose-response curve. (B) TB (20 μg/ml) was added to HeLa S3 and HEK-293 cells during HSV-1 adsorption (ADS) or after viral adsorption for the following 24 h (POST). HSV-1 was also preincubated with 20 μg/ml TB at 37°C, and the mixture was used to infect human cells for 24 h (TB/HSV-1, 37°C). Supernatants were subjected to a standard plaque assay to evaluate viral production. Data are means ± the SD from three independent experiments, each performed in duplicate (*, *P* < 0.05; **, *P* < 0.01; ***, *P* < 0.001 versus untreated sample).

On the basis of the obtained results, TB has emerged as a promising anti-HSV-1 agent. Thus, we tested its activity on two different human epithelial cells, HeLa S3 and HEK-293, already used for HSV-1 studies (21, 22). First, the potential cytotoxic effect of TB was evaluated by the MTT assay. As shown in Fig. 7A, no toxicity was observed at concentrations as high as 50 μg/ml TB. The IC_50_s were 93 and 84 μg/ml for HeLa S3 and HEK-293 cells, respectively. Viral replication was then assayed with or without TB. The results demonstrated that TB was also effective against HSV-1 replication in both human epithelial cell lines (Fig. 7B). In particular, the peptide reduced plaque formation by about 2 logs when it was added during the adsorption phase. The strongest effect (an ∼5-log reduction) was observed when the peptide was preincubated with HSV-1 virions for 1 h at 37°C, confirming virucidal activity of TB.

## DISCUSSION

We have demonstrated here for the first time that the AMP TB exerts significant anti-HSV-1 activity, mainly through its direct virucidal properties. Previous mechanistic studies on the antimicrobial effect of temporins against bacteria or fungi demonstrated that their activity is related to disruption of the microbial membrane (23, 24). Several authors have reported that certain defensins, cationic peptides that exert their antimicrobial activity through membrane permeabilization of the invading microorganism (25), also display antiviral activity against various enveloped DNA or RNA viruses, including HSV-1, HSV-2, cytomegalovirus, vesicular stomatitis virus, and influenza virus A/WSN (9, 26–29). However, these peptides are not able to inactivate two nonenveloped viruses: echovirus 11 and reovirus 3. The same authors suggested that the defensins interact with the viral envelope, causing lipid bilayer perturbation (27). Also, amphibian AMPs, such as dermaseptins, have been reported to inhibit HSV-1 and HSV-2 replication (30, 31). The mechanism of action seemed to be related to their characteristic cationic charge. Indeed, ionic interactions between positively charged amino acids present in the peptide sequence and the negatively charged sulfate and carboxyl groups in heparan sulfate of cell surface proteoglycans (cellular receptors for viral B and C glycoproteins) could block the attachment of the virus to the host cell (31). With regard to the temporins, Chincar et al. (19) showed the ability of the isoform A to inhibit channel catfish virus and frog virus 3 replication, although the mechanism of action remains to be determined. Here, we report that TB exhibited high anti-HSV-1 activity, particularly when preincubated alone with herpesvirus particles prior to cell infection, thus causing disruption of the viral envelope, as observed by TEM analysis. In comparison, TA did not affect HSV-1 envelope integrity. The differences in primary structure position of the single basic residue (Arg7 in TA and Lys10 in TB [see Fig. 6]) and the hydrophobicity of the two peptides (16) are factors that may account for the ability of TB to destroy the viral envelope in comparison to TA. This is in accordance with literature data showing a greater tendency for TB to destabilize both anionic and zwitterionic membranes (32). Note that viral envelope contains lipid rafts that have been reported to regulate surface charge and protein localization and to be responsible for the electrostatic interaction with cationic peptides (33, 34). Importantly, while the mode of action of AMPs against bacterial cells has already been well characterized and studied, their antiviral mechanism is not yet fully understood. Nevertheless, according to the proposed model of destabilization of influenza virus envelope, peptide molecules may insert into and disrupt the lipid bilayer after electrostatic interaction with the viral capsid. This would be followed by disruption of the viral envelope with leakage of viral components (35).

TB treatment also affected the attachment phase of HSV-1 cycle, but this inhibition was more modest with respect to TB/HSV-1 preincubation. The reason for this trend could be related to the weakly cationic nature of the peptide due to the presence of only one basic residue (lysine) in its sequence. However, despite the weak cationic character, the interactions between the peptide and the host cell surface might partially affect the virus attachment to the host cell. When cell monolayers were pretreated for 3 h with TB and then infected with HSV-1, no viral titer impairment was observed with respect to untreated cells, indicating that the inhibitory activity of TB is not due to its interference with the cellular receptors. Interestingly, when TB was added during the viral adsorption phase, the HSV-1 titer reduction was similar to that observed during the attachment assay, thus supporting the ability of the peptide to inhibit the very early stage of the viral multiplication cycle.

Furthermore, we also found out that administration of TB to the cells during the postinfection phase could inhibit HSV-1 infection. TB-induced HSV-1 inhibition was more pronounced when two consecutive doses of the peptide, i.e., during the viral adsorption and postinfection phases, were used. These data suggest that TB can also exert its antiviral activity by affecting one or more cellular targets that the virus exploits for promoting its life cycle. Indeed, it is known that HSV-1, like other viruses, manipulates host cell machinery and metabolism to its advantage (36–38). Some AMPs exert their antibacterial activity through additional targets regulating essential biochemical reactions, e.g., RNA, protein, or DNA biosynthesis and/or inhibition of cytosolic enzymes (39). Accordingly, in our experimental system, when TB was added as double doses (ADS+POST), we observed a strong reduction in ICPs and late viral protein expression, including gB. It is important to note that this glycoprotein, as well as others, is involved in the cell-to-cell spread of HSV-1 (20), and we found that TB is able to prevent spread from infected cells to neighboring uninfected cells. Therefore, we hypothesize that the peptide, by impairing gB expression, may interfere with HSV-1 spreading.

Due to the multiplicity of targets, AMPs are considered less likely to induce resistance mechanisms in microbes than conventional drugs (40). Regarding HSV-1, the available antiviral drugs to fight the viral infections are acyclovir and its derivatives, but, unfortunately, immunocompromised patients who require longer-term anti-HSV therapy can develop drug resistance (7). Thus, the search for new antiviral agents acting on both viral particles and cell pathways useful for the virus may control HSV-1 infection and bypass pharmacological resistance. In this scenario, TB can be considered a promising preventive or therapeutic antiherpetic agent, especially for topical usage. Of note, recent technological breakthroughs have sharply raised interest in the use of peptides as efficacious and safe new therapeutics (39, 41). This is confirmed by the increasing number of antitumor and/or antiviral AMPs, which is expected to lead to a shake up in their current scenario, moving such AMPs from clinical experimentation to the marketplace (42). Thanks to advances in nanotechnology, it is possible to conjugate peptides to nanocarriers in order to protect them from proteolytic degradation and prolong their bioavailability (43). In addition, the development of nanoformulations has contributed to optimize peptide delivery to the target site (44–47). The use of peptide-based drugs, mainly for the topical treatment of infections, is thus conceivable.

## MATERIALS AND METHODS

### Peptide.

Synthetic TB (LLPIVGNLLKSLL-NH2) was supplied at a purity of >98% by Selleck Chemicals, as previously reported (48). Briefly, the peptide was synthesized by standard Fmoc methodology and purified by reversed-phase high-pressure liquid chromatography on a semipreparative C_18_-bonded silica column (Kromasyl; 5 μm, 100 Å, 25 cm by 4.6 mm) using a gradient of acetonitrile in 0.1% aqueous trifluoroacetic acid at a flow rate of 1.0 ml/min.

### Cell culture and virus production.

African green monkey (Vero) cells were grown in RPMI 1640 medium supplemented with 10% heat-inactivated fetal bovine serum (FBS), 0.3 mg/ml l-glutamine, 100 U/ml penicillin, and 100 μg/ml streptomycin at 37°C in an atmosphere of 5% of CO_2_. Human embryonic kidney (HEK-293) cells and human cervix epithelial (HeLa S3) cells were grown in Dulbecco modified Eagle medium (DMEM) supplemented with 10% FBS, 0.3 mg/ml l-glutamine, 100 U/ml penicillin, and 100 μg/ml streptomycin. All reagents were purchased from Sigma-Aldrich. Virus production was performed as previously reported (49). Briefly, monolayers of Vero cells in 75-cm^2^ tissue culture flasks were infected with HSV-1 (strain F) at an MOI of 0.01. After 48 h at 37°C, the needed time to observe the virus-induced cytopathic effect, infected cells were harvested using three freeze-thaw cycles, cellular debris was removed by low-speed centrifugation for 10 min, and the virus titer was measured in the supernatants by a standard plaque assay, as described below. The titer of the virus preparation was 5 × 10^8^ PFU/ml. The virus was stored at –80°C until use.

### Cellular toxicity assays.

The cellular toxicity of TB was evaluated by a trypan blue (0.02% final concentration) exclusion assay (50). The viability and growth of Vero, HEK-293, and HeLa S3 cells were tested in *vitro* by an MTT assay, as previously reported (51, 52). Briefly, cell monolayers were incubated with TB at concentrations of 1, 10, 20, 50, 75, and 100 μg/ml (corresponding to 0.72, 7.2, 14.4, 36, 54, and 72 μM, respectively) in culture medium for 24 h; the medium was replaced with 50 μl of a 1-mg/ml solution of MTT in RPMI or DMEM without phenol red (Sigma-Aldrich). Cells were incubated at 37°C for 3 h, and then 100 μl of acidified isopropanol (0.1 N HCl in isopropanol) was added to each well. After a few minutes at room temperature to ensure that all crystals were dissolved, the plates were read using an automatic plate reader with a 550-nm test wavelength and a 690-nm reference wavelength. The CC_50_, defined as the drug concentration required for reducing the cell viability by 50%, was calculated by regression analysis of the dose-response curve generated from the data.

### In *vitro* HSV-1 infection.

Vero, HeLa S3, and HEK-293 cells were seeded in 24-well plates at a density of 1.5 × 10^5^ cells/ml and infected with HSV-1 at an MOI of 1. After incubation for 1 h at 37°C to allow virus adsorption to the host cells (adsorption phase), the medium was removed and replaced with fresh medium supplemented with 2% FBS, and the plates were maintained for 24 h at 37°C in an atmosphere of 5% CO_2_ (postinfection phase).

### Determination of viral yields.

The virus titer was measured using a standard plaque assay as previously described (53, 54). Briefly, supernatants of infected samples were used at different dilutions to infect monolayers of Vero cells. After 1 h at 37°C, the monolayers were washed, the medium was replaced with RPMI containing 2% CMC and 2% FBS, and the plates were maintained at 37°C for 48 to 72 h until plaque formation. The CMC solution was then removed, and the monolayers were fixed with cold methanol for 20 min at −20°C, washed with phosphate-buffered saline (PBS), and stained with a 0.5% crystal violet solution. The plaques were counted, and the virus titer was calculated as PFU/ml.

To assay antiviral activity, a plaque reduction assay was performed as previously described, with some modifications (55). Vero cells were seeded in 24-well plates at a density of 1.5 × 10^5^ cells/well. The next day, confluent cell monolayers were infected with HSV-1 at an MOI of 1 for 1 h at 37°C. The unabsorbed virus was subsequently removed by washing the cells three times with PBS, and the medium was replaced with RPMI 1640 containing 1% CMC, 2% FBS, and different concentrations of TB. After further incubation at 37°C for 36 to 48 h, the cells were fixed with cold methanol and stained with 0.5% crystal violet solution, and the viral plaques were counted. The concentration yielding a 50% reduction in plaque formation (IC_50_) was determined by regression analysis using GraphPad Prism v6.0 software by fitting a variable slope-sigmoidal dose-response curve.

### Pretreatment assay.

Vero cells were incubated with TB at 20 μg/ml for 3 h at 37°C, washed with PBS, and infected with HSV-1 at an MOI of 1 for 24 h. The supernatants of infected cells were used to determine the virus titer in a standard plaque assay.

### Time-of-addition assay.

TB (20 μg/ml) was added to a confluent monolayer of Vero cells infected with HSV-1 (MOI = 1) at different times of infection, as follows: (i) only during the viral adsorption period (1 h, 37°C); (ii) immediately after the adsorption period and maintained for the following 24 h; and (iii) during the viral adsorption period (1 h, 37°C) and immediately after the adsorption period for the following 24 h. HSV-1-infected HEK-293 and HeLa S3 cells were treated with TB (20 μg/ml) during the viral adsorption period (1 h, 37°C) or immediately after the adsorption period and maintained for the following 24 h. The supernatants from infected cells were used to determine the virus titer using a standard plaque assay, as described above.

### Attachment assay.

An attachment assay was performed as previously described (56), with some modifications. Briefly, Vero cells were incubated with a combination of TB and HSV-1 (MOI = 1) for 1 h at 4°C. The cells were then washed twice with PBS to remove both unattached virus and peptide and maintained for 1 h at 37°C in RPMI (adsorption phase). The cells were then washed three times with PBS, followed by incubation for 36 h at 37°C with RPMI 1640 containing 1% CMC and 2% FBS to perform the plaque reduction assay. Monolayers were then fixed with cold methanol and stained with 0.5% crystal violet solution, and the plaques were counted to determine the virus titer.

### Entry assay.

The entry assay was performed as previously reported (57), with some modifications. Briefly, confluent monolayers of Vero cells were infected with HSV-1 at an MOI of 1 for 1 h at 4°C. The cells were then incubated at a higher temperature (37°C) to maximize the penetration of virus in the presence or absence of TB. After 1 h, the supernatants were removed, and the cells were treated with PBS (pH 3) for 1 min to neutralize any remaining attached virus. After four washes with serum-free RPMI 1640, the cells were overlaid with medium containing 1% CMC and 2% FBS, followed by incubation at 37°C for 36 h to perform a plaque reduction assay. The monolayers were then fixed with cold methanol and stained with crystal violet solution, and the plaques were counted to determine the viral titer.

### Cell-to-cell spread assay.

The assay of the cell-to-cell spread of HSV-1 was performed as previously reported (58, 59), with some modifications. Briefly, Vero cells were seeded in 96-well plates and infected with HSV-1 at an MOI of 0.1 for 1 h at 37°C in the presence of TB (20 μg/ml, first dose). Cell monolayers were washed, and the medium was replaced with fresh medium containing TB (20 μg/ml, second dose) and 10 μg/ml anti-HSV-1 antibody (AbD Serotec) to neutralize HSV-1 particles released from infected cells and to ensure that HSV-1 infection was actually due to cell-to-cell virus spread. As controls, Vero cells were infected with HSV-1 and treated or not treated with neutralizing HSV-1 antibody or with TB. At 24 h postinfection, the cells were fixed with 4% paraformaldehyde in PBS for 15 min and permeabilized in 0.1% Triton X-100–PBS for 5 min at room temperature. After incubation with Odyssey blocking buffer for 1 h at room temperature, the cells were incubated with mouse anti-gB (Santa Cruz; 1:1,000 dilution in Odyssey blocking buffer) for 1 h at room temperature. After three washes, the cells were incubated with labeled secondary antibody IRDye 800 CW goat anti-mouse (LI-COR Biosciences, 1:1,000 dilution in Odyssey blocking buffer) for 1 h at room temperature (54). Finally, the plate was scanned on an LI-COR Odyssey infrared imaging system, and the relative fluorescence intensity of each well was read by using LI-COR Image Studio software developed for Odyssey analysis. The obtained images represented the foci of HSV-1 infection (green).

### HSV-1/temporin preincubation.

TB (20 μg/ml) was preincubated with HSV-1 (MOI = 1) at 37°C for 1 h, and the mixture was used to infect cellular monolayers. After viral adsorption for 1 h at 37°C, the plates were washed, and the medium was replaced with fresh medium containing 1% CMC and 2% FBS for 36 h at 37°C. The virus titer was evaluated as already described.

### Western blot analysis.

Infected Vero cells, treated or not treated with TB, were washed with PBS, recovered, and centrifuged at 770 × *g* for 10 min., The pellet was suspended in cold lysis buffer (10 mM Tris-HCl, 150 mM NaCl, 1 mM phenylmethylsulfonyl fluoride, phosphatase inhibitor mixture, 1% Triton X-100 [pH 7.4]; Sigma-Aldrich) and incubated on ice for 30 min. After centrifugation (10,000 × *g* for 30 min) the supernatants were collected and assayed to determine the protein concentration (Bradford method; Bio-Rad). Equivalent amounts of proteins were suspended in sodium dodecyl sulfate (SDS) sample buffer containing 100 mM 1,4-dithiothreitol (Sigma-Aldrich), boiled at 100°C for 10 min, separated by SDS-PAGE, and blotted onto nitrocellulose membranes for a Western blot assay. The membranes were blocked with 10% nonfat dry milk (Bio-Rad) in Tris-buffered saline–0.1% Tween for 1 h at room temperature. Primary antibodies (anti-herpes simplex virus 1/2 [AbD Serotec], raised against ICPs and late structural viral proteins; anti-gB [Santa Cruz Biotechnology]) were added at a final concentration of 1 μg/ml and maintained overnight at 4°C. Secondary antibodies were horseradish peroxidase-conjugated (Jackson ImmunoResearch). Blots were developed with ECL-Plus detection system (Thermo Scientific) and subjected to densitometric scanning.

### TEM.

Transmission electron microscopy (TEM) analysis was performed as described by Bultmann et al. (60) with some modifications. Briefly, for negative staining, a 10-μl drop of HSV-1 (1 × 10^6^ or 5 × 10^6^ PFU) containing TB or TA suspension, previously incubated at 37°C for 1 h, was placed on a carbon-coated grid for 30 s. Subsequently, the grid was washed with several droplets of phosphotungstic acid solution (2% [wt/vol], pH 6.8). The last droplet of staining solution was left for 10 s and then blotted with filter paper. The grid was then allowed to air dry before examination. For each condition, several grids were examined using a Philips CM 100 transmission electron microscope at an accelerating voltage of 100 kV (Microscopy Center, University of L'Aquila, L'Aquila, Italy). Ninety viral particles were examined.

### Statistical analysis.

Unpaired data were analyzed with a Student *t* test, and *P* values of <0.05 were considered significant. Data are presented as means ± the standard deviations (SD).
